# Remote Gait Type Classification System Using Markerless 2D Video

**DOI:** 10.3390/diagnostics11101824

**Published:** 2021-10-02

**Authors:** Pedro Albuquerque, João Pedro Machado, Tanmay Tulsidas Verlekar, Paulo Lobato Correia, Luís Ducla Soares

**Affiliations:** 1 Instituto de Telecomunicações, Instituto Superior Técnico, Universidade de Lisboa, Av. Rovisco Pais 1, 1049-001 Lisboa, Portugal; pedro.flores.albuquerque@tecnico.ulisboa.pt (P.A.); plc@lx.it.pt (P.L.C.); 2 Instituto de Telecomunicações, Instituto Universitário de Lisboa (ISCTE-IUL), Av. das Forças Armadas, 1649-026 Lisboa, Portugal; jpsmo11@iscte-iul.pt (J.P.M.); lds@lx.it.pt (L.D.S.); 3 Department of CSIS and APPCAIR, BITS Pilani, K K Birla, Goa Campus, Goa 403726, India

**Keywords:** assisted living, gait classification, pathology identification, remote diagnosis, web application

## Abstract

Several pathologies can alter the way people walk, i.e., their gait. Gait analysis can be used to detect such alterations and, therefore, help diagnose certain pathologies or assess people’s health and recovery. Simple vision-based systems have a considerable potential in this area, as they allow the capture of gait in unconstrained environments, such as at home or in a clinic, while the required computations can be done remotely. State-of-the-art vision-based systems for gait analysis use deep learning strategies, thus requiring a large amount of data for training. However, to the best of our knowledge, the largest publicly available pathological gait dataset contains only 10 subjects, simulating five types of gait. This paper presents a new dataset, GAIT-IT, captured from 21 subjects simulating five types of gait, at two severity levels. The dataset is recorded in a professional studio, making the sequences free of background camouflage, variations in illumination and other visual artifacts. The dataset is used to train a novel automatic gait analysis system. Compared to the state-of-the-art, the proposed system achieves a drastic reduction in the number of trainable parameters, memory requirements and execution times, while the classification accuracy is on par with the state-of-the-art. Recognizing the importance of remote healthcare, the proposed automatic gait analysis system is integrated with a prototype web application. This prototype is presently hosted in a private network, and after further tests and development it will allow people to upload a video of them walking and execute a web service that classifies their gait. The web application has a user-friendly interface usable by healthcare professionals or by laypersons. The application also makes an association between the identified type of gait and potential gait pathologies that exhibit the identified characteristics.

## 1. Introduction

Gait can be defined as the act of locomotion, involving periodic body movements, such as sequences of loading and unloading of the limbs [1]. The study and analysis of gait in a medical context can contribute to the diagnosis and monitoring of pathologies that affect people’s gait [2]. For this reason, the automatic classification of the type of gait is gathering interest, with many approaches already available in the literature [3,4]. Of these approaches, vision-based solutions appear to be especially interesting since image sequences can be captured with relatively simple setups, e.g., with a single 2D camera [5]. This enables the capture of gait data in a clinical environment or even at home, with most of the processing required to analyze the observed gait done remotely. A prototype based on this idea is proposed in this paper to enable the remote classification of people’s gait.

Most state-of-the-art vision-based systems for gait classification rely on deep learning strategies [6,7,8]. They involve the use of Convolutional Neural Networks (CNN), such as VGG-19 [9], pre-trained on the ImageNet [10], and fine-tuned using gait datasets. Fine-tuning requires relatively smaller datasets to adjust an existing CNN to perform better on a related problem. The quality of this adjustment and the expected results depend on the richness and suitability of the used datasets. However, most publicly available datasets containing different types of gait are captured from a limited number of healthy subjects simulating gait pathologies. Most datasets contain simulations because of the ethical and privacy concerns involved in sharing data from real patients. However, simulating pathologies with all its complexity is seldom correctly executed.

This paper presents a new gait dataset, GAIT-IT, containing 21 subjects and five types of gait, at two severity levels, simulated following instructions provided using an illustrative video, an oral explanation, and a short walking demonstration. The gait video sequences are captured in a professional studio with a chroma-keying background, resulting in a high contrast between the foreground and the background. These characteristics of the dataset are helpful for training a reliable gait type classification system.

The paper also presents a novel gait type classification system based on a CNN architecture. It drastically reduces the number of trainable parameters, compared to the state-of-the-art, thus having lower memory requirements and faster execution times. The proposed system is trained using the GAIT-IT dataset and tested using also a publicly available dataset containing simulations of the corresponding gait pathologies. The results suggest that the proposed system has significant generalization ability, as it can correctly associate available gait types with the corresponding pathologies. The results also highlight the effectiveness of the proposed system to operate in a relatively noisy acquisition setup of the GAIT-IST dataset, which was captured using a cell phone in an ‘at home’-like setting, with a wall as background and without particular care taken with the illumination, which came from a side window and the ceiling fluorescent lamps. Additionally, the distance from the camera to the subjects is different in the GAIT-IT and GAIT-IST datasets.

A third contribution of this paper is a web application for gait assessment. It is the prototype of a remote healthcare system, performing diagnosis by analyzing video sequences captured and uploaded from a cellphone or a personal computer. The web service identifies the type of gait and associates it to a possible gait related pathology. All computations are performed on the server, and the results are returned in a user-friendly manner, with images highlighting the parts of the gait that contribute more to the diagnosis.

### 1.1. Related Work

A rich characterization of gait information can be obtained through the use of different types of sensors, including [3]:

1. floor-based sensors;
2. wearable sensors;
3. vision-based sensors.

Floor-based sensors are used to detect ground reaction force [11], or the pressure exerted on the area under the foot [3]. It typically provides limited information for gait classification and the equipment used is restricted to constrained spaces. Wearable sensors are portable, allowing data acquisition of three-dimensional information related to walking patterns over long periods of time [4,12]. However, their performance can be influenced by the sensor placement. If sensor placement is not completed carefully, walking can become uncomfortable, which can then affect the quality of gait data acquired. Additionally, if an ‘at-home’ scenario is envisaged, for self-monitoring, then it is not guaranteed that sensors will be correctly applied, and the captured data may not be the intended type. In summary, sensor placement should always be completed under the supervision of trained professionals.

Vision-based systems have the advantage of being unobtrusive and not requiring a complicated cooperation of the subject. Marker-based systems are considered as the gold standard approach for gait analysis [13], using special markers placed on key body parts to track them and obtain kinematic features from the observed motion. However, these often require specialized personnel to ensure correct setup and calibration. On the other hand, a markerless approach can be more suitable for application in less constrained environments [14].

**Figure 1:**
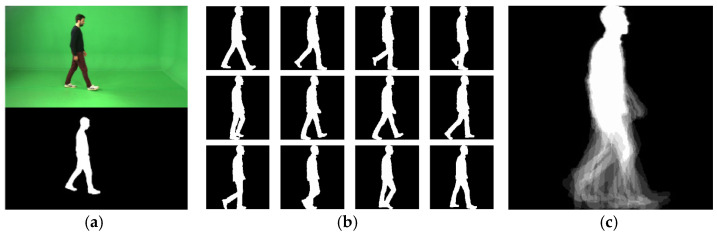
Example of (**a**) background subtraction, (**b**) binary silhouettes and (**c**) GEI.

Markerless vision-based systems for gait analysis typically follow a model-based or an appearance-based approach [15]. In the model-based approach, gait representations are created by fitting a model to the input sequence of images or silhouettes, using prior knowledge of the human body (structural model) or its motion (motion model) [16]. An example includes using two Kinect sensors with perpendicular viewing directions, acquiring both RGB and depth information to create a 3D model based on the movement of skeleton parts [17]. This model combines static features (e.g., distances between joints) and dynamic features (e.g., speed, stride length or the body’s center of mass movement). In the appearance-based approach, gait is represented without assuming prior knowledge of human motion. A sequence of binary silhouettes is typically obtained using background subtraction, as illustrated in Figure 1a. As long as a well performing background subtraction method is used, the resulting silhouettes are mostly free from background clutter and the influence of illumination changes. The desired gait representation can then be derived using the sequence of binary silhouettes. A widely used representation called the Gait Energy Image (GEI) [18] is obtained by averaging the cropped, normalized in size and horizontally aligned binary silhouettes belonging to a gait cycle, according to Equation (1).

(1) $$GEI\left(x,y\right)=\frac{1}{N}\overset{N}{\underset{i=1}{\sum }}B_{i}\left(x,\;y\right)$$

In Equation (1), *N* represents the number of frames in one (or multiple) gait cycle(s). *B_i_(x*, *y)* is a binary silhouette image, with *x* and *y* being pixel coordinates. The resulting GEI is a grey-level image implicitly representing, in a single image, the subject’s shape and motion along the gait cycle, as illustrated in Figure 1b,c. The GEI representation is robust against noise in individual frames.

**Figure 2:**
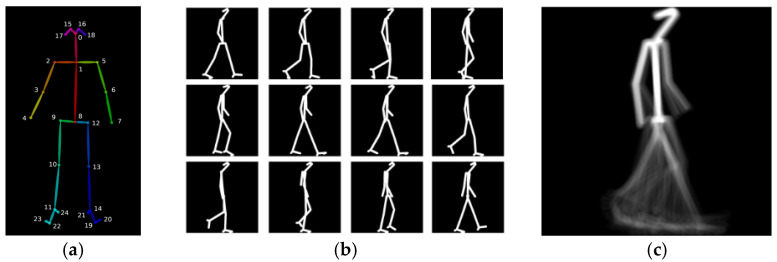
Example of (**a**) output of Open Pose [19], (**b**) skeleton images and (**c**) SEI.

A second representation considered for the presentation of results in this paper is the Skeleton Energy Image (SEI) [6], a hybrid between model- and appearance-based approaches—see Figure 2c. It starts by obtaining skeleton models for every available image of the walking person, using Open Pose [19]. Open Pose is a neural network trained to locate the positions of key joints of a human body on a 2D image, as illustrated in Figure 2a. With a skeleton image for each frame, the SEI can then be obtained with the same method used for GEI computation. The SEI was reported to achieve better pathological gait classification results than the GEI, as the SEI focuses on the dynamic movement characteristics and not on the physical constitution and clothing of a subject [6].

#### 1.1.1. Gait Classification Systems

Systems for the classification of gait types often use the gait representation directly, they compute a set of biomechanical features, or use a combination of both. For instance, the work reported in [20] describes two approaches, one using leg angles as features, and another one using the GEI. A set of normalized gait features is proposed in [21], including the step length, stance and swing phase durations, or the amount and broadness of limb movements, to quantify gait impairments. The last decade has witnessed the emergence of deep learning strategies for feature extraction in image recognition and classification with very good results, including gait analysis systems. The solution presented in [8] adopts the GEI for gait representation and uses the VGG-19 [9], pre-trained on a subset of ImageNet [10], for feature extraction. Transfer learning is used to repurpose the CNN for pathological gait classification, with the last layers of the VGG-19 network being trained using GEIs computed from the INIT dataset [21]. Linear Discriminant Analysis (LDA) was used for classification and the system’s performance is tested using two other pathological gait datasets: DAI [22] and DAI2 [20]. Another deep learning approach, also based on the VGG-19, is adopted in [6] for pathological gait classification, using both GEI and SEI gait representations. In this case, the pre-trained CNN is fine-tuned with data from the GAIT-IST dataset [6]. Other deep learning approaches include the use of Recurrent Neural Networks (RNNs) that are able to learn correlations between inputs in a time series, such as the application of a bidirectional Long-Short Term Memory (LSTM) [23] network for pathological gait classification based on sequences of lower limb flexion angles [7]. Given the good performance reported in the literature, this paper also considers a deep learning solution to perform gait type classification.

#### 1.1.2. Gait Datasets

Publicly available gait datasets are created either for biometric recognition, or for gait type classification. Datasets for recognition include subjects walking normally, possibly with some covariates such as different speeds, different types of shoes, different clothing or carrying different items. The purpose of gait type datasets is to capture sequences reflecting different kinds of impairments, notably to mimic the effects of some pathological conditions. Since sharing data from real patients raises ethical and data privacy issues, the publicly available impaired gait datasets are captured from healthy subjects simulating a selection of gait impairments. To the best of our knowledge, there are four gait impairment datasets publicly available, as listed below. All the sequences in these datasets are captured from a canonical viewpoint and recorded in controlled environments.

The DAI dataset [22] contains binary silhouettes of five subjects. It has 15 healthy gait sequences, and 15 sequences with random gait impairment simulations, for a total of 30 gait sequences. The subjects are captured walking over a distance of 3 m using both the RGB camera of a Kinect sensor and a smartphone.

The DAI2 dataset [20] also considers five subjects, but contains a total of 75 gait sequences. Each subject simulates four pathologies (Parkinson’s, diplegia, hemiplegia and neuropathy), as well as normal walking gait. Each condition was recorded 3 times, while walking along a distance of 8 m.

The INIT dataset [21] contains binary silhouettes of 10 subjects (9 males, 1 female), for a total of 80 sequences. Every subject is recorded 2twodifferent times, at 30 fps, capturing multiple gait cycles and simulating seven different gait impairments (in addition to a healthy gait sequence): (i) right arm motionless; (ii) half motion of the right arm; (iii) left arm motionless; (iv) half motion of the left arm; (v) full body impairments; (vi) half motion of the right leg; and (vii) half motion of the left leg.

The GAIT-IST dataset [6] considers 10 subjects, with a total of 360 gait sequences. The dataset includes the same four pathologies considered in DAI2, with two severity levels for each, two directions of walking, and two repetitions per subject, except for the normal gait. It is the largest pathological gait dataset publicly available. Video sequences were captured using a smartphone camera, with a resolution of 1280 × 720 pixels, mounted on a tripod at about 1.5 m above the ground and at a distance of about 4 m from the target.

Of the above datasets, some include impairments that are very easy to simulate, but which may not be directly related to any specific gait pathology. Other datasets include simulations of the gait pathologies, which are harder for healthy people to simulate. The proposed GAIT-IT dataset simulates different gait types that can be associated with known pathologies. Healthy volunteers were instructed on how to perform the simulations by watching detailed explanation videos, as well as personal interaction to clarify questions and see a short demonstration of the main walking characteristics related to the pathologies to simulate. GAIT-IT also doubles the number of subjects relatively to the largest publicly available dataset.

## 2. Materials and Methods

This paper presents three novel contributions:

1. proposal of a new, larger, gait type dataset: GAIT-IT;
2. a gait type classification system;
3. a remote diagnosing web application.

### 2.1. GAIT-IT Dataset

The proposed GAIT-IT dataset (available at http://www.img.lx.it.pt/GAIT-IT/ accessed on 3 September 2021) captures a larger number of subjects, with significantly more variations than the existing publicly available datasets. The sequences are captured at a higher quality and with a better contrast between the subject and the background. GAIT-IT is recorded in the professional studio of FCT|FCCN, Lisbon, Portugal (https://www.fccn.pt/en/colaboracao/estudio/ accessed date 25 July 2021), on two different days. The studio includes controlled artificial lighting and a green background, ideal for chroma-keying segmentation, resulting in high-quality sequences, free from background camouflage and other artifacts. Two professional 4K video cameras are used to capture synchronized gait sequences, one with a side view, at approximately 3 m from the target, and the other with a front/rear view, at about half a meter from the walking start position. Both cameras stood on tripods at 1.75 m from the ground.

**Figure 3:**
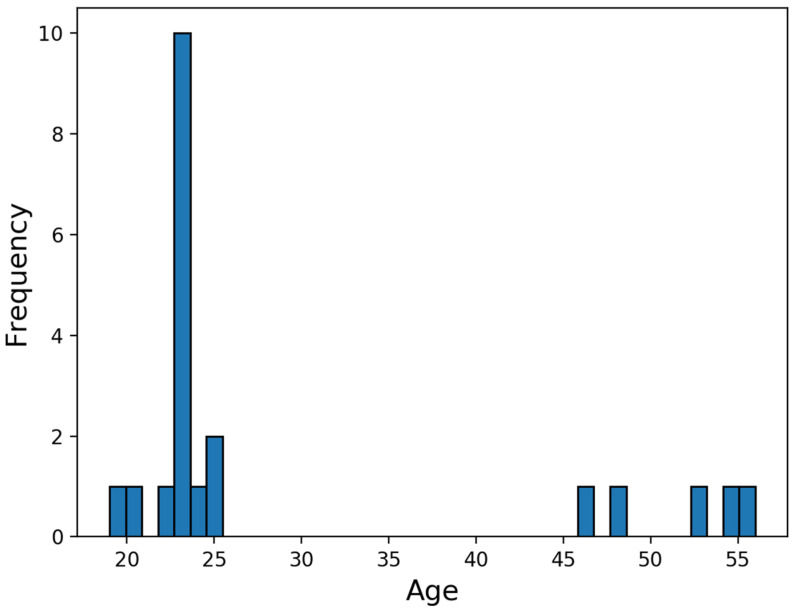
Subjects’ age distribution.

The GAIT-IT dataset contains simulations of five different types of gait. For each type, except normal, two levels of severity are captured. The subjects provide four gait sequences per severity level. This corresponds to a subject walking twice from left to right and from right to left, from the side view. The sequences are captured on two different days where 21 volunteers (19 males and 2 females) between the age range of 20 to 56 years participated, with a mean of 29.5 and a standard deviation of 11.6—see Figure 3. Thus, GAIT-IT dataset includes a total of 828 gait sequences. Having some subjects captured on different days, allows intra-subject variations in the simulations. Before capturing the sequences, the subjects are instructed on how to simulate the various gait types and severity levels, as summarized below [24].

The scissor gait commonly associated with diplegia affects both sides of the body. A subject adopts a forward leaning posture and walks by dragging both feet in a circular motion. For the second severity level the overall bending is accentuated, along with leg and arm movements.

The spastic gait commonly associated with hemiplegia affects only one side of the body. The leg is dragged in a circular motion, with a broader reach for the second severity, while the right arm remains still and held close to the waist, or flexed against the chest in the second severity level.

The steppage gait commonly associated with neuropathy leads to foot drop. Subjects tend to lift their knees higher than normal to avoid dragging their toes on the floor. In the second severity level, the lift of the leg and the forward swing are exaggerated.

The propulsive gait commonly associated with Parkinson’s diseases is characterized by a stooped posture, with both arms held close to the chest and the lower limbs flexed and rigid. Subjects are asked to attempt simulating general and erratic body shaking while taking small and relatively fast steps. The second severity level involves an overall exaggeration of these symptoms.

The captured sequences are processed to produce four different representations:

1. sequence of binary silhouettes;
2. sequence of skeletal images;
3. GEIs;
4. SEIs.

GEI and SEI representations are obtained for each gait cycle, as well as for the complete set of gait cycles available per sequence. The spatial dimension of the produced gait representations is 224 × 224 pixels. The binary silhouettes are cropped and overlapped following Equation (1) to obtain the gait representations. All representations consider a framerate of 10 fps. The main steps for obtaining the gait representations are as follows.

The extraction of binary silhouettes relies on chroma-keying segmentation. A frame containing only the background is represented in the HSV color space and the histograms of the hue (H), saturation (S) and value (V) components are computed. Then, all pixels in gait sequences with HSV values outside the background range are classified as belonging to the walking subject. Finally, a morphological filtering operation is applied to remove noise. A sample result is presented in Figure 1a. Skeleton computation relies on locating key anatomical parts in the gait images, using the Open Pose [19] software, which uses a multi-stage CNN to automatically detect a total of 135 body, hand, facial and foot key points in each frame of a video, operating in real-time. In the current implementation, only 25 key points corresponding to the body are captured, as illustrated in Figure 2a. The GEIs and SEIs are computed following Equation (1). An example of the gait representations obtained from the GAIT-IT dataset is illustrated in Figure 1c (GEI) and Figure 2c (SEI).

### 2.2. Gait Type Classification System

**Figure 4:**
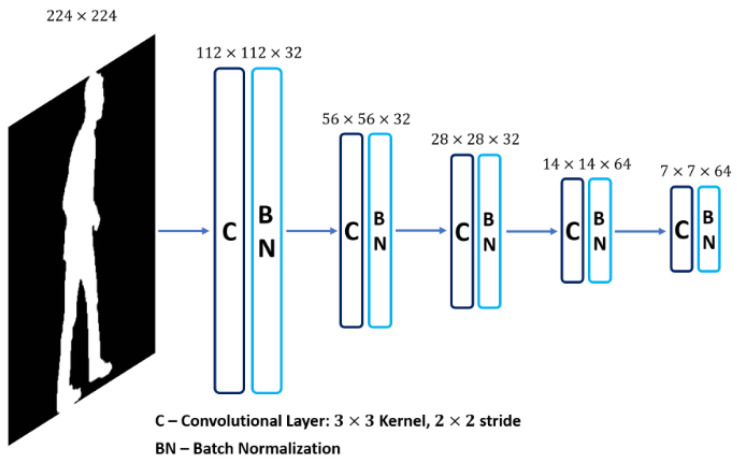
The proposed gait type classification CNN architecture.

The state-of-the-art vision-based systems for gait type classification rely on deep learning using pre-trained CNNs, which are then fine-tuned by transfer learning with task-specific datasets. This strategy is employed as most gait type datasets contain a limited amount of training data. Since the proposed GAIT-IT dataset provides a considerable increase in the amount of data available for training, rather than fine-tuning a complex network, this paper proposes a novel lightweight CNN, specifically trained to perform gait type classification. The architecture of the proposed CNN is illustrated in Figure 4.

The proposed system accepts a GEI or SEI as an input, which is processed using five convolutional layers. This option follows the type of architectures adopted in the Kaggle MNIST challenge [25,26], which also process binary images. As in the architectures of popular CNNs, such as VGG-16, the proposed system adopts a 3 × 3 filter size and a stride of 2 for the convolutional layers. A total of 32 feature maps, or filters, is considered in the first convolutional layer, being doubled for the last two layers. Each convolutional layer is followed by batch normalization, to adjust and scale the outputs to have a mean value close to 0 and a standard deviation close to 1. Bounding the values that pass between layers helps to stabilize and speedup the training process.

To perform classification, the features computed by the final convolutional layer are flattened and passed on to a fully connected neural network, consisting of two dense layers with a dropout [27] of 0.5 between them. The first dense layer has 512 units and the second layer has five units, corresponding to the five considered gait types, with a softmax activation function to output class probabilities. The proposed system is trained using categorical cross entropy and the Adam algorithm [28], with the Nesterov momentum variation [29]. The learning rate is set to 0.001.

### 2.3. A Remote Diagnostic Web Application Prototype

This paper also proposes the prototype of a system that allows remote gait diagnosis. It could assist healthcare professionals to identify patients requiring immediate attention and further examination, as well as monitor the evolution of existing gait pathologies, without the need of physical interaction with the patient. The usefulness of such a system is made more evident under the COVID-19 pandemic.

The proposed remote diagnostic web application runs the proposed gait type classification system on its server. It can be access by issuing HTTP requests to the web service. The web interface allows uploading a video sequence or a compact gait representation, notably a GEI or a SEI. It then executes the web service and returns the results to be presented in way that can be easily interpreted by the user.

The web application offers two different modes of operations:

1. basic mode;
2. advanced mode.

**Figure 5:**
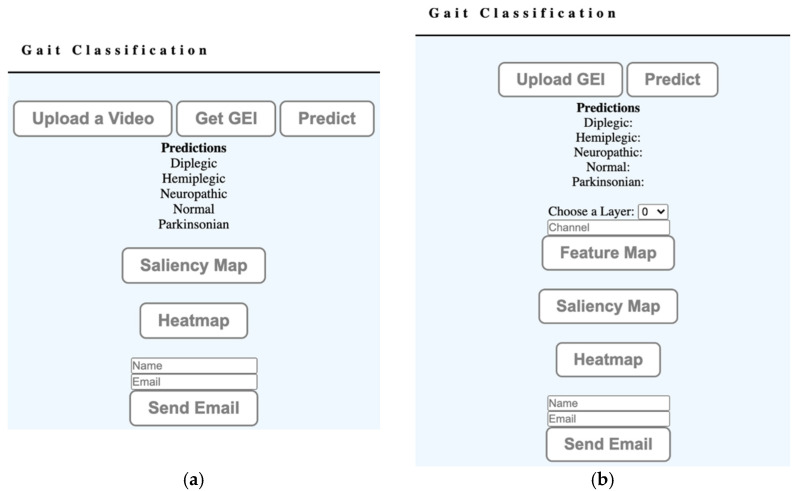
Web service user interface modes of operation: (**a**) basic, (**b**) advanced.

**Figure 6:**
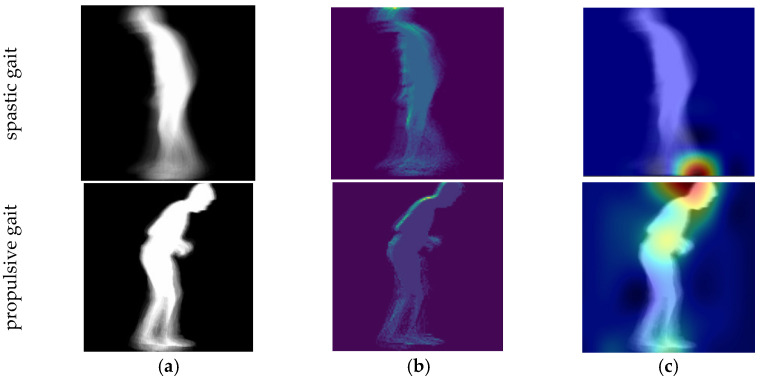
Output of the visualizer (**a**) GEI, (**b**) saliency feature map, (**c**) class activation map.

The basic mode is a simple interface to be used in a clinical environment or at home. It assumes a simple setup which involves filming a subject using a 2D camera, e.g., using a cellphone’s camera. The user interface, illustrated in Figure 5a, allows the user to upload the video, and the web application generates a GEI (or SEI) representation. The gait representation is processed by the web service using the proposed gait type classification system, which checks if the identified features could be associated to a specific gait pathology. The user can then visualize the parts of the body that contributed to the diagnostic using a saliency representation [30] and class-activation maps (grad-CAM) [31]. Figure 6 illustrates results for two different types of gait, suggesting that spastic gait is identified by the characteristic movement of the feet, while propulsive gait is identified by the type of feet movement and the bending of the spine. The diagnostic can optionally be sent to a specified e-mail address. The interface is designed to remotely obtain a preliminary diagnostic, and to help visualize the body motions that deviate from a healthy gait.

The advanced mode uses the interface illustrated in Figure 5b, providing additional details for those interested in analyzing the operation of the classification system. It allows users to visualize the feature maps generated by specified convolutional layers. The visualization of the feature maps can offer a low-level insight into the training process. It also allows users to directly upload GEIs or SEIs to the system.

The remote diagnostic web application prototype can be further improved to allow training the classification system with different gait representations. The visualization of detailed features supported by the advanced user interface mode can provide an important insight to understand the operation of the classification system.

## 3. Results

The proposed gait type classification system is evaluated using a 10-fold cross-validation protocol on the GAIT-IT dataset. To emphasize the proposed system generalization capability, a second set of evaluation results considers the proposed system trained on the GAIT-IT dataset and tested on GAIT-IST. To compare the proposed system performance with the state-of-the-art, the systems presented in [6,8] are considered here for benchmarking. These systems use a solution based on VGG-19, pre-trained on Imagenet, and then fine-tuned using GEIs [6] and SEIs [8]. Those systems are re-implemented and fine-tuned using the GAIT-IT dataset, for fairness of the presented comparisons.

First, the proposed and the state-of-the-art systems [6,8] are evaluated using a 10-fold cross-validation protocol. The GAIT-IT dataset is split into training and test sets, where the subjects in each set are mutually exclusive. The test set for each fold is defined as *V_k_* = {*S_i_*, *S_i_*_+1_, *S_i_*_+2_}, where *i = 2 × k − 1*, *k* is the fold iteration and *S_i_* represents one of the 21 subjects. This arrangement ensures the use of every subject in the test set at least once, thus reducing training bias. The cross-validation results are presented in Table 1. Table 2 additionally compares the neural network model size and the execution times for the proposed and the state-of-the-art [6,8] systems, using a personal computer equipped with an AMD Ryzen 7 1700X processer, 32 GB RAM and a GTX 1070 GPU with 8 GB.

Training and testing a classification system using the same dataset can raise the issue of overfitting. To address this issue, a cross-dataset evaluation is additionally performed using the GAIT-IST [6] and the proposed GAIT-IT datasets. This second set of evaluations are conducted by training the proposed and the state-of-the-art [6,8] systems using all available subjects from the GAIT-IT dataset, and then testing the gait type classification systems using all the available subjects from the GAIT-IST dataset. It should be noted that GAIT-IST dataset acquisition setup is significantly different from GAIT-IT, with acquisition performed using a cell phone camera, under a ceiling light. Table 3 reports the obtained classification accuracy results, while Table 4 reports the corresponding confusion matrix. Training with GAIT-IST or DAI2 was not considered as those datasets are significantly smaller and some of the available silhouettes contain segmentation errors. The other publicly available datasets discussed in Section 1.1.2 were not considered because they include simulations of limb movement impairments, rather than gait types, and their size is small.

## 4. Discussion

The average classification accuracy obtained using 10-fold cross validation, reported in Table 1, suggests that the proposed system’s performance, achieving a classification accuracy of 93.4% and 92.6% on GEI and SEI gait representations, respectively, is equivalent to the state-of-the-art [6,8]. However, it should be noted that the proposed system has a much lower computational complexity, due to the significantly smaller number of trainable parameters and the consequent reduction of static and dynamic memory needed to store and execute the system—see Table 2. The proposed system, represented in hdf5 [32] file format, is 83 times smaller than the state-of-the-art VGG-19 system. The proposed gait type classification system also executes significantly faster, which is of great importance for considering the deployment of a diagnostics web service to operate over the Internet. Table 2 also reports training and execution time for the proposed and the state-of-the-art [6,8] systems, showing that the proposed system operates 15 times faster during training and six times faster when processing a request.

The cross-dataset results reported in Table 3 suggest that the proposed system generalizes better than the state-of-the-art VGG-19 systems [6,8]. The proposed system improves the average classification accuracy by 3.4% and 1.3% using GEIs and SEIs, respectively. A possible explanation for this increase may be that the deeper CNN architecture requires significantly more data for fine-tuning, and to avoid overfitting to the seen training data. Thus, the shallower CNN model of the proposed gait type classification system appears to be more suitable for operation with datasets with limited training data.

Table 4 reports results of classification across five different types of gait when testing with the GAIT-IST dataset [6]. This test assumes an association between the gait types simulated in the novel GAIT-IT dataset (scissor, spastic, steppage, propulsive and normal), and the gait pathologies simulated in the GAIT-IST dataset (diplegic, hemiplegic, neuropathic, Parkinsonian, healthy), with the results obtained confirmed to be a reasonable assumption.

To further analyze the proposed system’s performance, the confusion matrix presented in Table 4 highlights the prediction errors made by the proposed system. From these results it can be inferred that normal gait is the easiest to classify, with a classification accuracy of 99%., while the scissor gait is the most difficult to classify, with a classification accuracy of 87%. This can be due to the scissor gait GEIs’ showing a similar appearance to spastic and propulsive GEIs, as these three types of gait involve a limited leg movement. The spastic gait performs slightly better with an average classification accuracy of 89%. The distinct walking pattern of steppage gait allows the system to achieve an average classification accuracy of 97%. Propulsive gait achieved the next best classification accuracy of 95% as it involves a stooped posture along with the restricted leg movements. Finally, it can be concluded that the proposed gait type classification system can be used to successfully identify gait impairments from 2D video sequences, which may be captured using the pervasive smartphone devices (as considered in the GAIT-IST dataset).

## 5. Conclusions

This paper presents the prototype of a web application for remote gait diagnostic system. The application, to be used over the Internet, implements a web service that executes a gait type classification system on the server, returning results to be reported using a user-friendly graphical interface. The novel gait type classification system is based on a shallow CNN architecture, whose performance is equivalent to the state-of-the-art classification systems [6,8], while showing two distinct advantages:

1. The proposed deep learning model is 83 times smaller than the one considered by state-of-the-art solutions [6,8]. This reduces the memory requirements and improves the execution time, which is significant when operating over the Internet;
2. The shallower network model achieves a better fit using the GAIT-IT dataset, which contains data from only 21 subjects, as confirmed by the cross-database test results. This is significant as the proposed web application accepts video sequences captured under different conditions and environments.

The paper also presents GAIT-IT dataset, containing 828 gait sequences, captured from 21 subjects simulating five different types of gait. The sequences were captured using two synchronized cameras, capturing both the sagittal and frontal views. The dataset contains silhouettes, skeletons, GEIs and SEIs.

Since this work focuses on the sagittal view, future work can consider the integration of frontal view analysis. The combination of orthogonal viewpoints can result in more discriminative features, leading to an improved classification system. Furthermore, different deep network architectures can be considered to explore the temporal nature of gait. The web application prototype is presently hosted as a web service in a private network, and after further development, e.g., to allow training the system with additional types of gaits and other gait representations, it might be made publicly available. The model is also be released in GitHub (https://github.com/jpsmachado/Gait-WebApp.git accessed date 23 July 2021).

Another possible future direction can include extending the GAIT-IT dataset to incorporate sequences from real patients. Since all the existing publicly available datasets, including GAIT-IT dataset, are composed of simulations, testing the proposed system with real patients will allow further validation of its performance in classifying gait pathologies.

## Figures and Tables

**Table 1:** Cross-validation results obtained using the GAIT-IT dataset.

Gait Classification System	Input	Accuracy (%)
Fine-Tuned VGG-19 [8]	GEI	94.0
Fine-Tuned VGG-19 [6]	SEI	93.6
Proposed system	GEI	93.4
Proposed system	SEI	92.6

**Table 2:** Number of parameters, storage space, training and execution time (milliseconds) required by the VGG-19 [6,8] and the proposed systems.

Gait Classification System	Parameters	Size (Mb)	Execution Time (ms)	
			Train	Test
Fine-Tuned VGG-19 [6,8]	139,330,565	558.4	15	6
Proposed system	1,684,421	6.8	1	1

**Table 3:** Cross-dataset results obtained using GAIT-IT for training and GAIT-IST for testing.

Gait Classification System	Input	Accuracy (%)
Fine-Tuned VGG-19 [8]	GEI	86.4
Fine-Tuned VGG-19 [6]	SEI	85.1
Proposed system	GEI	89.8
Proposed system	SEI	86.4

**Table 4:** Confusion matrix for the proposed gait type classification system representing an average score of GEI and SEI inputs (%).

Predicted Class						
**True Class**	**Gait Type**	**Scissor** **(Diplegic)**	**Spastic** **(Hemiplegic)**	**Steppage (Neuropathic)**	**Normal** **(Healthy)**	**Propulsive (Parkinsonian)**
	Scissor	87	7	0	0	5
	Spastic	9	89	2	0	0
	Steppage	0	2	97	1	0
	Normal	0	0	0	99	0
	Propulsive	5	0	0	0	95

## Data Availability

The GAIT-IT dataset is available at http://www.img.lx.it.pt/GAIT-IT/ (accessed on 3 September 2021).
